# The kingdom of the prolyl-isomerase Pin1: The structural and functional convergence and divergence of Pin1

**DOI:** 10.3389/fcell.2022.956071

**Published:** 2022-08-30

**Authors:** Yew Mun Lee, Deryn En-Jie Teoh, Kay Yeung, Yih-Cherng Liou

**Affiliations:** Department of Biological Sciences, Faculty of Science, National University of Singapore, Singapore, Singapore

**Keywords:** Pin1 orthologs, WW domain, peptidyl-prolyl *cis/trans* isomerase (PPIase), phosphorylation, structure-function of Pin1

## Abstract

More than 20 years since its discovery, our understanding of Pin1 function in various diseases continues to improve. Pin1 plays a crucial role in pathogenesis and has been implicated in metabolic disorders, cardiovascular diseases, inflammatory diseases, viral infection, cancer and neurodegenerative diseases such as Alzheimer’s, Parkinson’s and Huntington’s disease. In particular, the role of Pin1 in neurodegenerative diseases and cancer has been extensively studied. Our understanding of Pin1 in cancer also led to the development of cancer therapeutic drugs targeting Pin1, with some currently in clinical trial phases. However, identifying a Pin1-specific drug with good cancer therapeutic effect remains elusive, thus leading to the continued efforts in Pin1 research. The importance of Pin1 is highlighted by the presence of Pin1 orthologs across various species: from vertebrates to invertebrates and Kingdom Animalia to Plantae. Among these Pin1 orthologs, their sequence and structural similarity demonstrate the presence of conservation. Moreover, their similar functionality between species further highlights the conservancy of Pin1. As researchers continue to unlock the mysteries of Pin1 in various diseases, using different Pin1 models might shed light on how to better target Pin1 for disease therapeutics. This review aims to highlight the various Pin1 orthologs in numerous species and their divergent functional roles. We will examine their sequence and structural similarities and discuss their functional similarities and uniqueness to demonstrate the interconnectivity of Pin1 orthologs in multiple diseases.

## Introduction

Extensive studies during the past 25 years since Pin1’s discovery led to an evolutional discovery on the post-phosphorylation regulation through the elucidation of Pin1’s structure, its involvement in diseases, and the identification of Pin1 orthologs conservation across various species. Pin1 is part of the parvulin family of peptidyl-prolyl isomerases (PPIases), and it contains two distinct domains: the N-terminal WW domain and the catalytic C-terminal PPIase domain (Lu et al., 1996). In 1989, Hanes et al. (1989) discovered the first Pin1 orthologue in *Saccharomyces cerevisiae* as protein Ess1 and revealed the importance of Ess1 in growth and division. Subsequently, in 1996, Lu et al. (1996) identified and characterised the human Pin1 (hPin1) protein, demonstrating its homology to Ess1 and their shared activity in regulating cell growth. Apart from the baker’s yeast *S. cerevisiae*, Pin1 orthologs are identified in numerous other species documented in the UniProt database (Consortium, 2021). Researchers primarily focused their studies of Pin1 orthologs on model organisms: namely, the fruit fly *Drosophila melanogaster*, the nematode *Caenorhabditis elegans*, the mouse mammalian model organism *Mus musculus*, the zebrafish *Danio rerio*, and the plant species *Arabidopsis thaliana* and *Malus domestica* (Maleszka et al., 1996; Fujimori et al., 1999; Yao et al., 2001; Bitomsky et al., 2013; Del Rosario et al., 2015). The nature of homology is such that there are variations between orthologs of different species, and Pin1 is no exception. Therefore, when studying these Pin1 orthologs, questions of transferability arise: How do differences in the structure of Pin1 orthologs affect their functions within different species? Does the variation between species affect hPin1 disease research? What are the degrees of structural and functional convergence and divergence between Pin1 orthologs?

With these questions in mind, this review aims to describe the well-characterised orthologs of Pin1, explore the structure-function relationship of Pin1 in different species, and discuss how variations in Pin1 orthologs impact investigating Pin1’s involvement in human diseases.

## Pin1 orthologs

**FIGURE 1:**
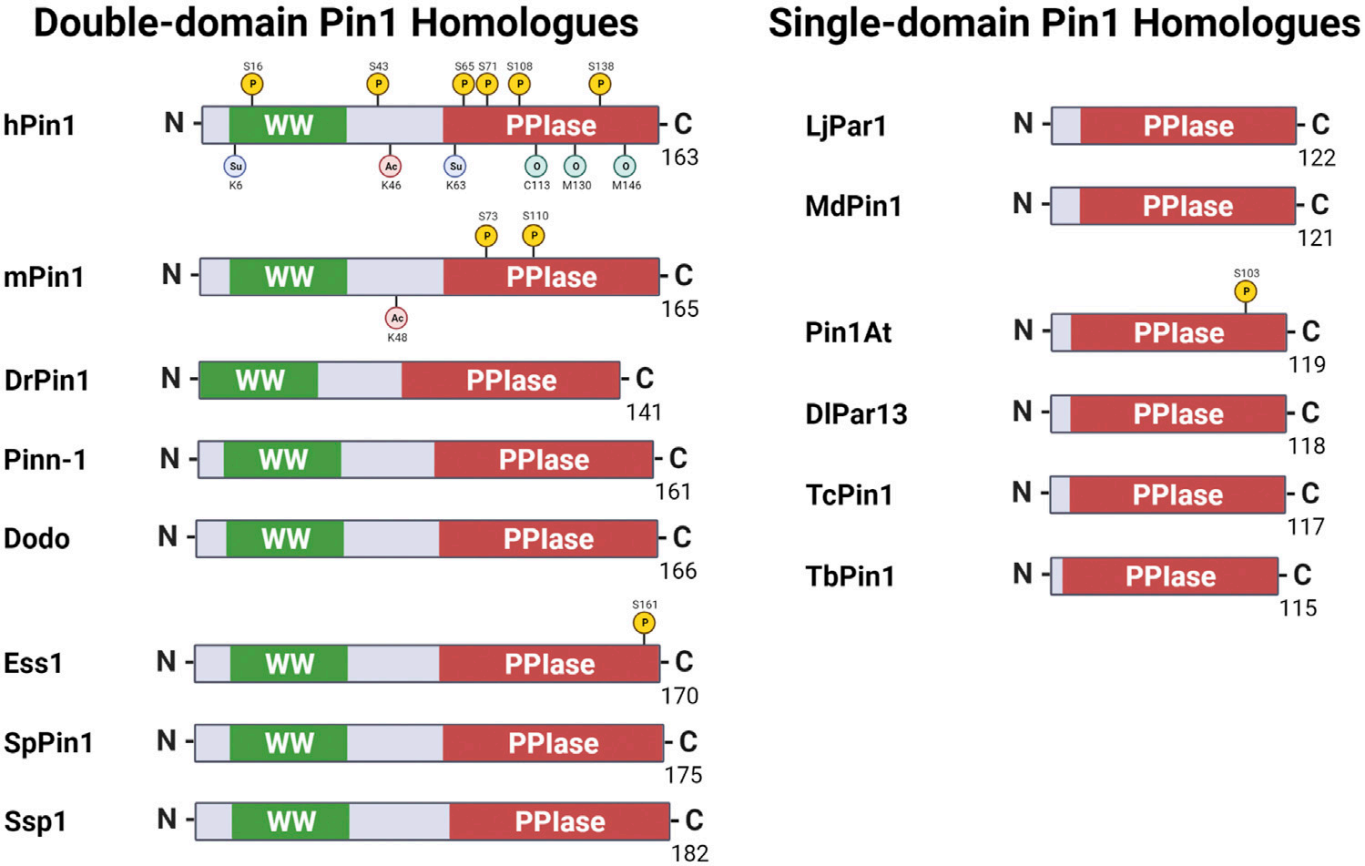
Pin1 orthologs across various species. Pin1 orthologs possess either the WW and peptidyl-prolyl isomerase (PPIase) domains or just the single PPIase domains. hPin1: Human Pin1; mPin1: Mouse Pin1; DrPin1: *Danio rerio* Pin1; Pinn-1: *Caenorhabditis elegans* Pin1; Dodo: *Drosophila melanogaster* Pin1; Ess1: *Saccharomyces cerevisiae* Pin1; SpPin1: *Schizosaccharomyces pombe* Pin1; Ssp1: *Neurospora crassa* Pin1; LjPar1: *Lotus japonicus* Pin1; MdPin1: Malus domestica Pin1; Pin1At: *Arabidopsis thaliana* Pin1; DlPar13: *Digitalis lanata* Pin1; TcPin1: *Trypanosoma cruzi* Pin1; TbPin1: *Trypanosoma brucei* Pin1. P: phosphorylation; Su: sumoylation; Ac: acetylation; O: oxidation. Created with BioRender.com.

Human Pin1 function is modelled mainly through model organisms, such as *C. elegans* and *M. musculus*. It is thus reasonable that, despite the hundreds of Pin1 orthologs discovered (National Library of Medicine (US), 2004), the Pin1 orthologs found in model organisms such as yeast and mice are the most well-studied. This review section briefly describes some key Pin1 orthologs (Figure 1) and highlights the functional similarity and variation of such orthologs across species.

In 1996, Lu and colleagues elucidated the hPin1 protein for the first time using the yeast-two-hybrid screen to identify proteins that interact with a Ser/Thr protein kinase NIMA (never in mitosis gene A). Due to the interaction of Pin1 with NIMA, the group revealed the essential role of hPin1 in cell cycle progression. Pin1 is a member of the parvulin family, and its discovery marked the first human PPIase to be involved in mitotic regulation. Human Pin1 shows a 45% sequence similarity to the known PPIase Ess1, found in *S. cerevisiae*. Furthermore, Pin1 could complement *ess1* yeast mutants and rescue the cell-cycle-defective phenotype of *ess1* yeast mutants, showing hPin1 functional similarity and strong homology to Ess1 (Lu et al., 1996).

In 1989, 7 years before hPin1’s discovery, a study by Hanes et al. (1989) identified the *S. cerevisiae* protein Ess1 (termed Ptf1 in their paper), which was later characterised as the first eukaryotic member of the parvulin family of PPIases, named after the parvulin protein found in *Escherichia coli* (Rahfeld et al., 1994; Hani et al., 1995). Ess1 contains 172 amino acids and is essential in regulating cell growth, cell division, and mitotic progression (Hanes et al., 1989). The loss in function or overexpression of *ess1* in yeast can lead to growth defects, aberrant morphology, or cell death (Hanes et al., 1989). Another study by Hani et al. (1999) also demonstrated that Ess1/Ptf1 functions in pre-mRNA 3′-end processing, as mutations of the protein led to defects in pre-mRNA 3′-end formation.

On top of Ess1 from the baker’s yeast *S. cerevisiae*, the fission yeast *Schizosaccharomyces pombe* and the fungus *Neurospora crassa* also contain Pin1 orthologs SpPin1 and Ssp1, respectively (Kops et al., 1998). Huang et al. (2001) demonstrated that overexpression of SpPin1 can rescue the cell cycle defects caused by *ess1* yeast mutants, suggesting that SpPin1 is a positive regulator of Cdc25 and Wee1 mitotic proteins (Huang et al., 2001). Ssp1 was first identified by Kops et al. (1998) and is unique from its other eukaryotic members of the parvulin family, such as Pin1. Ssp1 is more abundant in *N. crassa* than hPin1 in human cells. Physiologically, Ssp1 is also localised to the nucleus and the cytoplasm, unlike hPin1, which is mainly localised to the nucleus. Based on the Human Protein Atlas, hPin1 is expressed in all tissue (Uhlén et al., 2015). The highest abundance of hPin1 is in the neuronal cells of the central nervous system, with subcellular localization to both the cytoplasm and nucleus. Moreover, Ssp1 also contains a conserved polyglutamine stretch, with unknown function, between the WW and PPIase domain. The authors also found Ssp1 to be a *cis/trans* isomerase and a potent mediator for protein folding.

In 1995, Maleszka et al. (1996) identified the *dodo* gene, encoding a 166 amino acids protein in *D. melanogaster*, as an Ess1 orthologue. Dodo showed 44% amino-acid identity to Ess1/Ptf1, and it displayed functional interchangeability by being able to rescue *ess1* *S. cerevisiae* knockout mutants (Maleszka et al., 1996). This observation implies that Dodo, like Ess1, functions in cell cycle regulation; however, unlike Ess1, Dodo showed an additional role in regulating the MAPK signal transduction during oogenesis (Hsu et al., 2001).

The Pin1 orthologue in the nematode *C. elegans* is known as Pinn-1. Pinn-1, containing 161 amino acids, inhibits DAPK-mediated excitotoxicity and neurodegeneration and regulates the spatiotemporal expression of neuronal ankyrin *via* alternative pre-mRNA processing (Liou et al., 2002; Del Rosario et al., 2015). Pinn-1 is also involved in heat and cold stress responses (Fasseas et al., 2012). Pinn-1, unlike its orthologs in *S. cerevisiae*, *D. melanogaster*, and *M. musculus*, has not been well-studied in its role in the cell cycle. However, its role in the pre-mRNA processing mechanism may be similar to that in *S. cerevisiae* as described by the study of Hani et al. (1999) that we have highlighted previously in this section.

The mPin1 protein in mice bears a ∼90% similarity to hPin1. Uchida’s group reported that loss of mPin1 in mice developed normally but had defectives in entering the cell cycle from G0 arrest, consistent with the cellular function of Pin1 in regulating the cell cycle (Fujimori et al., 1999). Further studies in mice revealed various cell-proliferative abnormalities, including decreased body weight and testicular and retinal atrophies. In female *pin1* null mice, the breast epithelial compartment was retarded and failed to proliferate during pregnancy. Remarkably, abnormal phenotypes, including retinal hypoplasia and mammary gland impairment, resemble the phenotypes in cyclin D1-deficient mice (Liou et al., 2002). Cellular assays also confirmed that mPin1 directly binds to and positively regulates cyclin D1 activity (Liou et al., 2002). Subsequently, it was reported that mPin1-deficient mice display a wide range of phenotypes, including induction of Neu/Ras-mediated mammary epithelial cell transformation, protection against endotoxin shock in microbial infection and regulation of skeletal muscle fusion during myogenesis and muscle regeneration (Ryo et al., 2002; Magli et al., 2010; Akiyama et al., 2011; Islam et al., 2018).

An important landmark in the study of mPin1 is centred on its role in Alzheimer’s disease (AD) (Pastorino et al., 2006). Previously, in 1999, Lu et al. (1999a) demonstrated that hPin1 localises to neurofibrillary tangles (NFTs) in neurons in human AD brain tissues. The authors found that hPin1 was trapped in the insoluble fraction consisting of the NFTs compared to the soluble fractions from AD brain tissue. Contrastingly, hPin1 was found primarily in the soluble fraction compared to the insoluble fraction of normal human brain tissues. Moreover, it was demonstrated that hPin1 binds specifically to phosphorylated tau protein at position T231 preceding Pro (pT231-P). However, hyperphosphorylation of tau in AD led to the loss of tau binding to microtubules, causing the formation of NFTs. The binding of hPin1 to pT231-P of tau protein can restore tau’s ability to bind to microtubules, thus restoring the microtubule assembly function and preventing NFTs. In the context of AD, the authors suggested that the hyperphosphorylated tau overwhelms the availability of hPin1 to bind to phosphorylated tau, leading to the formation of NFTs and sequestering the availability of soluble hPin1 for essential mitotic functions. Collectively, these drive the manifestation of AD. Significantly, a serine or threonine residue preceding a proline (S/T-P), the specific binding site of Pin1, is the most frequently phosphorylated motif in AD (Lu et al., 2002; Lu et al., 2003; Lu, 2004; Lu and Zhou, 2007; Iqbal et al., 2016; Wang et al., 2020).

Significantly, mPin1 knocked-out mice are the first mouse models to show tau-related and beta amyloid Aβ pathology from a single gene deletion (Liou et al., 2003; Pastorino et al., 2006; Lu and Zhou, 2007; Lee et al., 2011). The study by Liou et al. (2003) was the first of its kind to demonstrate that the single gene deletion for *pin1* in the mouse can lead to neurodegenerative phenotypes (Preuss and Mandelkow, 1998). The authors found that *pin1* ^−/−^ mice developed several age-dependent phenotypes, similar to tau transgenic mice (Lewis et al., 2000; Allen et al., 2002). In addition, degeneration of neurons was also observed. On the molecular level, *pin1* ^−/−^ mice led to the accumulation of MPM-2 epitopes commonly observed in AD and related disorders (Preuss and Mandelkow, 1998; Husseman et al., 2000; Lu et al., 2003). The *pin1* ^−/−^ mice also showed an increase in the level of the total phosphorylation of tau, with an apparent molecular weight 68 kDa, being hyperphosphorylated with NFT conformations. Accompanying the accumulation of MPM-2 epitopes and tau phospho-epitopes was the decrease in phosphatase activities towards phosphorylated a serine or threonine residue preceding a proline (i.e., pS/T-P motif). The authors also observed the formation of endogenous tau filaments in *pin1* ^−/−^ mice, similar to that in tau transgenic mice (Allen et al., 2002).

In a follow-up study by Lim et al. (2008), the authors examined the effects of mPin1 on the protein stability of wild-type tau and P301L tau. Transgenic (Tg) mice overexpressing human P301L tau are known to develop robust tauopathy phenotypes and are frequently used as an AD animal model (Lewis et al., 2000; Götz et al., 2007; Banerjee et al., 2022). The authors found that mPin1 overexpression promotes the degradation of phosphorylated tau (pT231-P), with mPin1 knockout leading to hyperphosphorylation of tau (pT231-P), inhibition of tau degradation, and neurodegenerative phenotypes. Interestingly, for P301L tau mutants, the function of mPin1 was reversed. Overexpression of mPin1 increased the hyperphosphorylation of tau, inhibiting tau degradation and inducing neurodegenerative phenotypes.

In contrast, mPin1 knockout promotes the degradation of phosphorylated tau. The authors suggested that P301L tau mutation led to an increased protective *trans* conformation of pT231-P tau, and mPin1 overexpression led to the acceleration of *trans* to *cis* isomerisation. The knockout of mPin1preserved the *trans* conformation and facilitated the degradation of P301L tau. In 2012, Nakamura et al. (2012) crossed *pin1* ^*−/−*^ mice with tau-Tg mice and demonstrated increased *cis* pT231-P tau and decreased *trans* pT231-P tau levels, further supporting that mPin1 suppresses tau-related neurodegeneration in mice. Besides using Pin1 mice models in studying taupathology in AD, Pastorino et al. (2006) also demonstrated that *pin1* ^−/−^ mice affect amyloid precursor protein (APP) processing in mouse brains with overexpressed APP. mPin1 knockout increased levels of toxic insoluble Aβ peptides Aβ42 in an age-dependent manner. These Aβ42 peptides were mainly found in multivesicular bodies of neurons with Aβ plaques. These studies in mPin1 mice models are seminal to the understanding of the role of Pin1 in neurodegenerative diseases such as AD.

Interestingly, Pin1 orthologs also exist in plants, not to be confused with the auxin efflux carrier component 1, also termed PIN1 (Gälweiler et al., 1998). The first plant Pin1 ortholog discovered in *Arabidopsis thaliana*, known as Pin1At, has a 53% similarity to the hPin1 protein’s PPIase catalytic domain (Landrieu et al., 2000). Although Pin1At lacks the WW domain found in animal Pin1 orthologs, it displays a similar mechanism of binding to pS/T-P sites and catalysing the *cis/trans*-isomerisation reaction around the proline residue (Landrieu et al., 2000). Additionally, a plant study by Yao et al. (2001) discovered that Pin1 proteins in *A. thaliana* (Pin1At) and *M. domestica* (MdPin1) both display the ability to rescue the phenotype of *ess1* mutants in *S. cerevisiae*, and that MdPin1 expression was associated with cell division. Subsequently, a year later, Metzner et al. (2001) identified another Pin1 orthologue in the plant, *Digitalis lanata* (DlPar13). Similar to Pin1At and MdPin1, DlPar13 also rescued the phenotype of *ess1* mutants in *S. cerevisiae*. Fast forward to 2009, Kouri et al. (2009) identified the Pin1 orthologue in *Lotus japonicus* (LjPar1), with sequence similarity to Pin1At (81.5%) and hPin1 (51.7%). As with all the known plant orthologs, they possess only the PPIase domain. Like DlPar13, LjPar1 is localised to both the nucleus and the cytoplasm. The authors also found that LjPar1 is upregulated during the later stages of nodule development, similar to other parvulins involved in organismal development. Taken together, it is clear that there is a functional similarity between plant Pin1 and animal Pin1 orthologs.

Although the enzymatic functions of the plant Pin1 orthologs were reported at the time, its physiological role was still unknown. Our group was the first to demonstrate that Arabidopsis Pin1, Pin1At, can control the flowering time of *A. thaliana*. Pin1At mediates the flowering of *A. thaliana via* its interaction with pS/T-P motifs of the MADS-domain proteins SOC1 (suppressor of overexpression of CO1) and AGL24 (agamous-like 24) and catalyses their *cis/trans* isomerisation (Wang et al., 2010). Pin1At regulates phosphorylated AGL24 by increasing its stability in the nucleus. Upregulation of Pin1At promotes flowering by accelerating the isomerisation of pS/T-P motifs in AGL24 and SOC1 and enhancing their stability, whereas depletion of Pin1At decelerates the isomerisation and delays the flowering. This finding not only reveals an essential regulatory mechanism in plant development regulated by Pin1At, but also sheds light on identifying Pin1At substrates and its relevant biological process in plants. This study also provides evidence that prolyl *cis/trans* isomerases are evolutionarily conserved in plants and animals.

Moreover, our subsequent study also discovered that Pin1At regulates root gravitropism (Xi et al., 2016). We showed that Pin1At binds to the pS/T-P motifs of PIN1 (auxin efflux carrier component 1) to accelerate its *cis/trans* isomerisation and modulates PIN1 polar localisation in the root stele cells under the governance of PID (AGC kinase PINOID) and PP2A (protein phosphatase 2A) phosphatase, with a similar mechanism in animals, regulated by the upstream kinase (i.e., MAPK) and downstream phosphatase (PP2A). Interestingly, depletion of Pin1At suppresses root gravitropic phenotypes of *pp2aa* and *35S::PID*, while overexpression of Pin1At affects root gravitropic responses and enhances the *pp2aa* gravitropic phenotypes. Our studies further demonstrated the diverse role of Pin1 orthologs and suggested an evolutionarily conserved prolyl *cis/trans* mechanism in plants and animals.

Pin1 orthologs in the plants are not the only ones that possess only the PPIase domain. In 2007, Erben et al. (2007) identified a Pin1 orthologue in the protozoan parasite *Trypanosoma cruzi* (TcPin1), with a 40% amino acid sequence identity to the PPIase domain of hPin1. Similarly, in 2010, our group identified a Pin1 orthologue in the other parasite *Trypanosoma brucei* (TbPin1), containing 115 amino acids of a 12 kDa protein, with 52.3% identity to the hPin1 (Goh et al., 2010). As with the plant Pin1 orthologs, both TcPin1 and TbPin1 possess the single PPIase domain without the WW domain. Furthermore, TcPin1 and TbPin1 can rescue the impaired temperature sensitivity in *ess1* mutant yeast.

**FIGURE 2:**
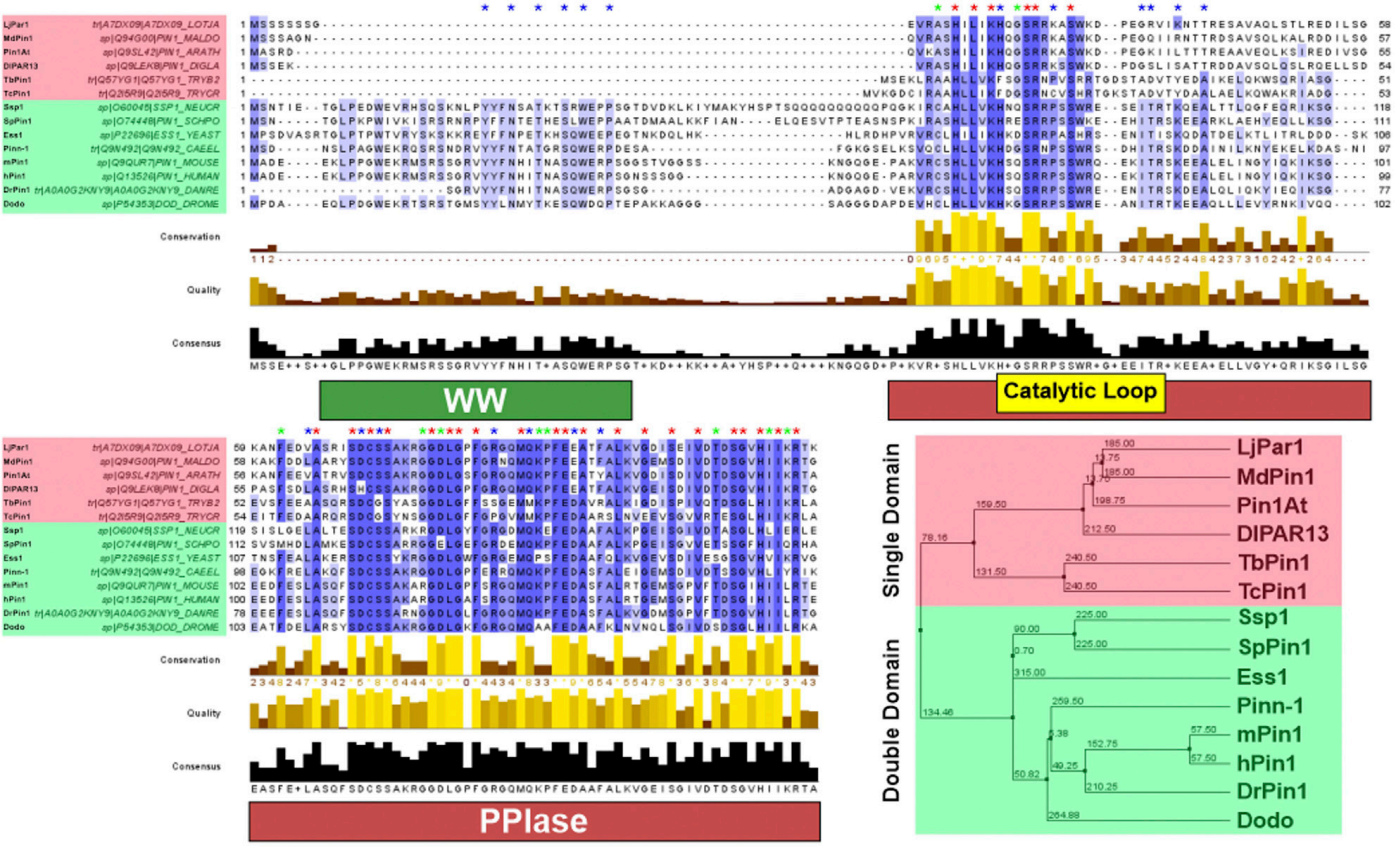
Pin1 sequence conservation across species. Pin1 orthologs highlighted red have only the PPIase domain; Pin1 orthologs highlighted green have both the WW and PPIase domains. Blue asterisk above the sequence indicates conserved amino acid only in Pin1 orthologs with double domain; green asterisk indicates conserved amino acid only in Pin1 orthologs with single domain; red asterisk indicates conserved amino acid in all Pin1 orthologs. The purple highlight of the Pin1 orthologs’ sequences indicates the degree of conservation across all Pin1 orthologs. A darker shade of purple demonstrates highly conserved amino acid across all Pin1 orthologs in that specific position, while decreasing shade of purple demonstrates reduced conservation. Amino acid sequences of each Pin1 ortholog are obtained from UniProtKB and processed using Jalview 2.11.2.1. Sequence alignment was done using the ClustalWS algorithm with default settings, and the hierarchical tree was done using BLOSUM62 algorithm based on average distance. The distances are indicated in the tree. hPin1: Human Pin1; mPin1: Mouse Pin1; DrPin1: *Danio rerio* Pin1; Pinn-1: *C. elegans* Pin1; Dodo: *D. melanogaster* Pin1; Ess1: *S. cerevisiae* Pin1; SpPin1: *S. pombe* Pin1; Ssp1: *Neurospora crassa* Pin1; LjPar1: *L. japonicus* Pin1; MdPin1: Malus domestica Pin1; Pin1At: *A. thaliana* Pin1; DlPar13: *D. lanata* Pin1; TcPin1: *T. cruzi* Pin1; TbPin1: *T. brucei* Pin1. The conservation histogram (top) reflects the conservation of the physicochemical properties of the amino acids, and absolutely conserved residues (max score 11) have a yellow asterisk “*”, and columns where physicochemical properties are conserved (score 10) have a yellow “+”; less conserved positions are shown in darker colours with decreasing score. The quality histogram (middle) reflects the likelihood of observing a mutation in any particular column of the alignment based on the BLOSUM62 matrix scores (for each column, the sum of the ratios of the two BLOSUM62 scores for a mutation pair, and each residue’s conserved BLOSUM62 score, are normalised and plotted on a scale of 0–1). The consensus histogram (bottom) reflects the percentage of the modal residue per column, and the consensus sequence logo is shown for conserved regions (“+” denotes non-conserved residues and “-” denotes gap residues).

From the examples of Pin1 orthologs highlighted in this review section, it is clear that Pin1 is highly conserved across species (Figure 2). It has orthologs in prokaryotes, animals, plants and even parasites. This high level of conservation alludes to the essential role that Pin1 plays in the cell. Indeed, *ess1* or *ptf1* null mutations in yeast led to a lethal phenotype (Hanes et al., 1989; Hani et al., 1995); however, it should also be noted that *pin1* mutant mice can still develop and grow despite some abnormalities in the phenotype in adulthood (Fujimori et al., 1999; Liou et al., 2003). This difference in phenotype shows the second point highlighted by this section, that there is functional variation between the orthologs, despite their high levels of similarity. As such, the next section of this review addresses the structure/function relationship of Pin1, which also explores the structural variation of Pin1 orthologs.

**FIGURE 3:**
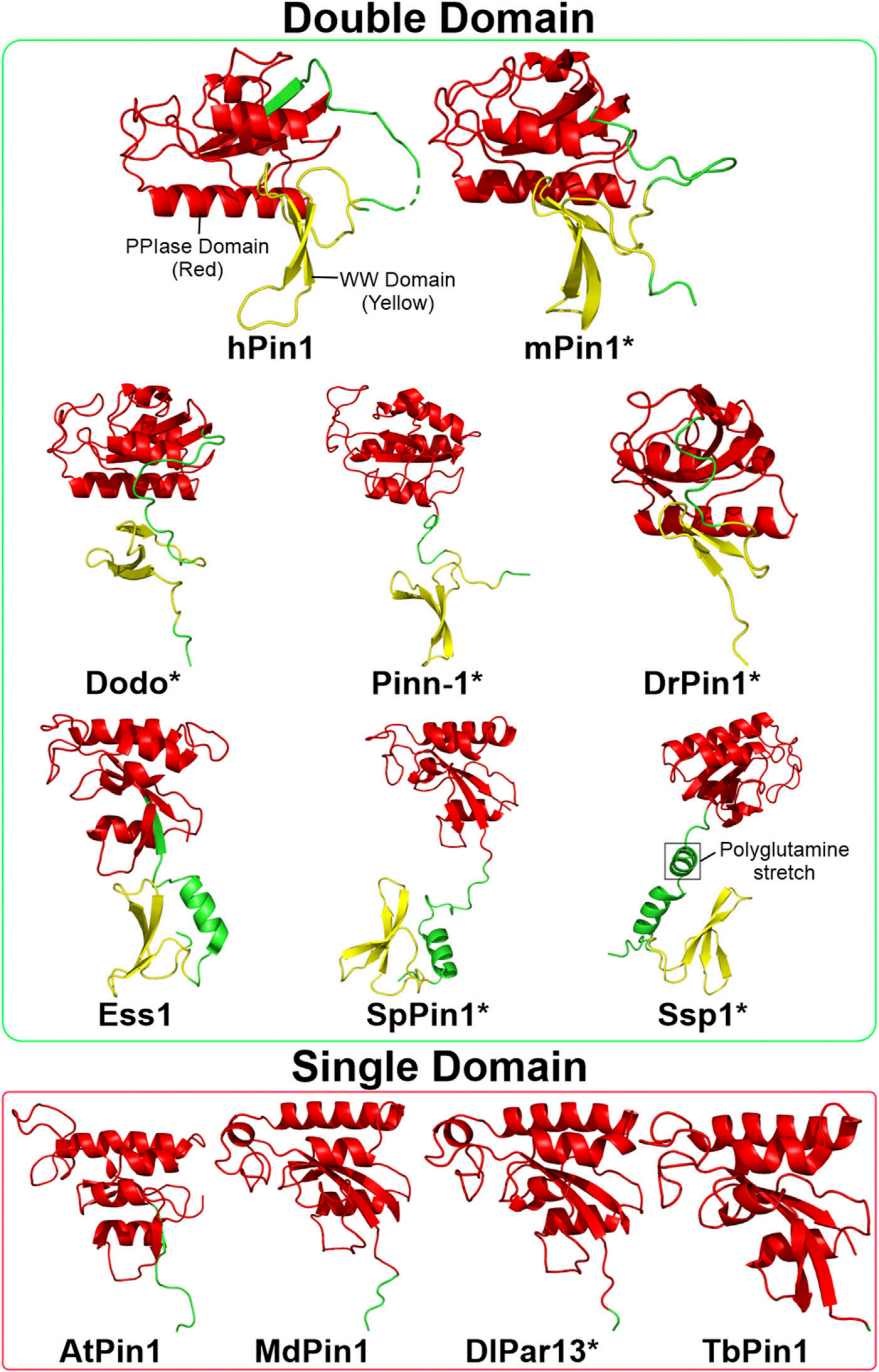
Structures of Pin1 orthologs across various species. The green box contains Pin1 orthologs with both WW and PPIase domains; the red box contains Pin1 orthologs with only the PPIase domain. Asterisks indicate that the protein structure is predicted using AlphaFold (Jumper et al., 2021). The linking region between the WW and PPIase domains of Ssp1 contains a conserved polyglutamine stretch. Red: PPIase domain; green: linking region; yellow: WW domain. Structures are obtained from UniProtKB and processed using PyMOL 2.5 software. hPin1: Human Pin1; mPin1: Mouse Pin1; DrPin1: *D. rerio* Pin1; Pinn-1: *C. elegans* Pin1; Dodo: *D. melanogaster* Pin1; Ess1: *S. cerevisiae* Pin1; SpPin1: *S. pombe* Pin1; Ssp1: *N. crassa* Pin1; LjPar1: *L. japonicus* Pin1; MdPin1: Malus domestica Pin1; Pin1At: *A. thaliana* Pin1; DlPar13: *D. lanata* Pin1; TcPin1: *T. cruzi* Pin1; TbPin1: *T. brucei* Pin1.

## Pin1 structure-function relationship

While Pin1 was first discovered in 1996 Lu et al. (1996), Ranganathan et al. (1997) reported the protein’s complete X-ray crystal structure a year later. Pin1 has two structural domains: the N-terminal WW domain (residues 1–39) and the catalytic C-terminal PPIase domain (residues 45–163). Furthermore, it contains a putative nuclear localisation signal (NLS), consistent with the observation associated with the nuclear speckle (Lu et al., 1996; Ranganathan et al., 1997). Subsequently, in 2009, Lufei and Cao demonstrated that hPin1 did contain an NLS within the PPIase domain and is required for hPin1 interaction with importin alpha five of the nuclear import machinery (Lufei and Cao, 2009). In this section, the review will explore the specific elements within these two domains that contribute to its binding and catalytic mechanism and the differences in structure between the Pin1 orthologs highlighted in the previous section.

The WW domain of Pin1 is similar to the WW domains found in many other proteins, including the transcriptional regulator protein YAP (yes-associated protein 1), the ubiquitin E3 ligase NEDD4 (neuronal precursor cell-expressed developmentally downregulated 4), and the skeletal muscle dystrophin (Sudol et al., 1995; Staub et al., 1996; Rentschler et al., 1999). The WW domain contains a conserved pair of tryptophan (W) residues, mediating protein-protein interactions. In all three of the above WW domain-containing proteins, the WW domains bind to proline-rich motifs (such as XPPXY and PPXT motifs) and contribute to their respective catalytic activities (Sudol et al., 1995; Staub et al., 1996; Rentschler et al., 1999). Drawing from this observation, researchers suggest that the WW domain of Pin1, given its high level of similarity to the WW domains of these various other proteins, also binds proline-rich motifs, thus contributing to the substrate-binding activity of Pin1 (Ranganathan et al., 1997). Indeed, subsequent studies demonstrated that Pin1’s WW domain specifically mediates its selectivity towards pS/T-P motifs (Rotin, 1998; Lu et al., 1999b; Ingham et al., 2005). The Pin1 WW domain is part of the Group IV WW domains group, which binds to pS/T-P motifs in a phosphorylation-dependent manner (Lu et al., 1999b). Structural analysis of Pin1 WW domain binding to a phosphorylated RNA-polymerase C-terminal domain (CTD) peptide revealed several critical residues in the domain that mediates this binding. The three crucial residues of Ser16, Arg17, and Tyr23 specifically bind the pSer residue of the CTD peptide *via* hydrogen bonding. Furthermore, Tyr23 and Tyr34 clamp the target site of the CTD peptide and its neighbouring residues *via* their aromatic rings. Notably, the binding of the WW domain to the CTD peptide led to an open conformation in the PPIase domain, which promotes the binding and activity of Pin1 (Verdecia et al., 2000).

The PPIase domain is responsible for the catalytic activity of Pin1, and it does so through three central regions within this domain: a basic triad, a hydrophobic pocket and a catalytic tetrad, all of which are in close vicinity to the protein’s active site (Ranganathan et al., 1997). The Lys63, Arg68, and Arg69 residues form the basic triad, and it binds to multivalent anions, such as phosphate or sulphate ions. In the context of Pin1, researchers proposed that this triad mediates the substrate specificity by selecting pS/T-P residues. Secondly, the hydrophobic pocket, comprising the residues Phe134, Met130, and Leu122, binds and stabilises the proline residue of the target peptide. Finally, the catalytic tetrad contains the residues Cys113, His59, His157, and Ser154, and they coordinate the *cis/trans*-isomerisation of the substrate (Ranganathan et al., 1997). The catalytic process of *cis/trans* isomerisation involves a non-covalent twisted amide mechanism. The Cys113 residue presents a negative charge to the carbonyl oxygen atom of the substrate, which destabilises the peptide bond between the pSer/Thr and the Pro residues in a stretch conformation. This destabilisation yields a twisted amide transition state stabilised by neighbouring hydrogen bonds and eventually shifts the peptide bond from a *cis* conformation to a *trans* conformation (Behrsin et al., 2007; Mercedes-Camacho et al., 2013).

Traditionally, the model of Pin1 activity is that the WW domain is responsible for protein specificity and the PPIase domain for catalysis. Indeed, research has shown that the PPIase domain alone cannot bind to Pin1 substrates, while the WW domain alone binds to Pin1 substrates in a phosphorylation-dependent manner (Lu et al., 1999b). However, experimental data showed that overexpression of the Pin1 PPIase domain alone could rescue the phenotype of *ess1*/*ptf1* temperature-sensitive mutants (Zhou et al., 2000). Furthermore, AtPin1 (or Pin1At) and MdPin1, both of which lack the WW domain, can rescue *ess1* mutants (Yao et al., 2001). These observations suggest some levels of ability by the PPIase domain to recognise, bind to, and act on the target peptides. A review by Lee and Liou describes the interplay between these two domains and highlights several potential binding models between Pin1 and substrate peptides. However, the community seems to have no consensus regarding one favoured binding model. The reason might be that there is not enough research on the conformational changes of the Pin1 upon binding to the substrates. This complexity between the WW and PPIase domains in substrate binding may explain the difficulty in elucidating a comprehensive overview of Pin1’s binding mechanism (Lee and Liou, 2018).

In concluding this review segment, it is clear that hPin1 has been well-studied concerning its structure. However, many questions remain unanswered due to its substrate recognition and binding complexity, particularly in the binding mechanism. The structural comparison between animal and plant Pin1 orthologs is of considerable interest since plant orthologs lack the WW domain but can complement *ess1* mutants (Zhou et al., 2000). Is the binding mechanism conserved between plants and animals, or is there a divergence wherein plants bind to *ess1* targets *via* a different mechanism? Additionally, the lack of structural data of Pin1 orthologs such as Dodo and Pinn-1 prevents comprehensive structural comparisons between animal Pin1 orthologs. Further research could be conducted in this field to characterise the variations in binding mechanism across Pin1 orthologs.

## Pin1 function and involvement in disease

The preference of Pin1 in binding to phosphoproteins highlights its role in regulating phosphorylation, a reversible post-translational modification that controls protein stability and activity. As such, Pin1 acts as a molecular switch in the cell, controlling the fate of its target phosphoproteins. Research shows that the isomerisation of target proteins by Pin1 affects downstream signalling pathways, as reviewed by Lu et al. (2007) and Liou et al. (2012). These reviews illustrate the phenomenon that *cis/trans* isomerisation affects protein recognition. In other words, enzymes such as ubiquitin ligases, kinases, and phosphatases can selectively target proteins in either a *cis* or *trans* conformation, indicative of Pin1’s role in regulating cellular signalling pathways (Liou et al., 2012). Indeed, Pin1 has a highly diverse role, regulating the activity of proteins involved widely in various cellular processes, including cell cycle, transcription, cell fate and development, and apoptosis (Hanes et al., 1989; Fujimori et al., 1999; Morris et al., 1999; Hsu et al., 2001; Liou et al., 2002; Atchison et al., 2003; Sorrentino et al., 2003; Xu et al., 2003; Ghosh et al., 2013). Pin1 is involved in many cellular diseases, such as cancer, neurodegenerative disease, viral infections, and metabolic disease, as highlighted in a review by Li et al. (2021) (Lu et al., 1999a; Wulf et al., 2002; Liou et al., 2003; Ryo et al., 2006; Lee et al., 2009; Lim et al., 2011; Nakatsu et al., 2011; Ghosh et al., 2013; Narita et al., 2013; Nakatsu et al., 2015; Nakatsu et al., 2017; Chen et al., 2018; Cheng and Tse, 2018; Nishi et al., 2020; Yu et al., 2020). A comprehensive review by Zannini et al. (2019) has also provided the importance of Pin1 regulation in many signalling pathways and biological processes leading to oncogenesis.

**FIGURE 4:**
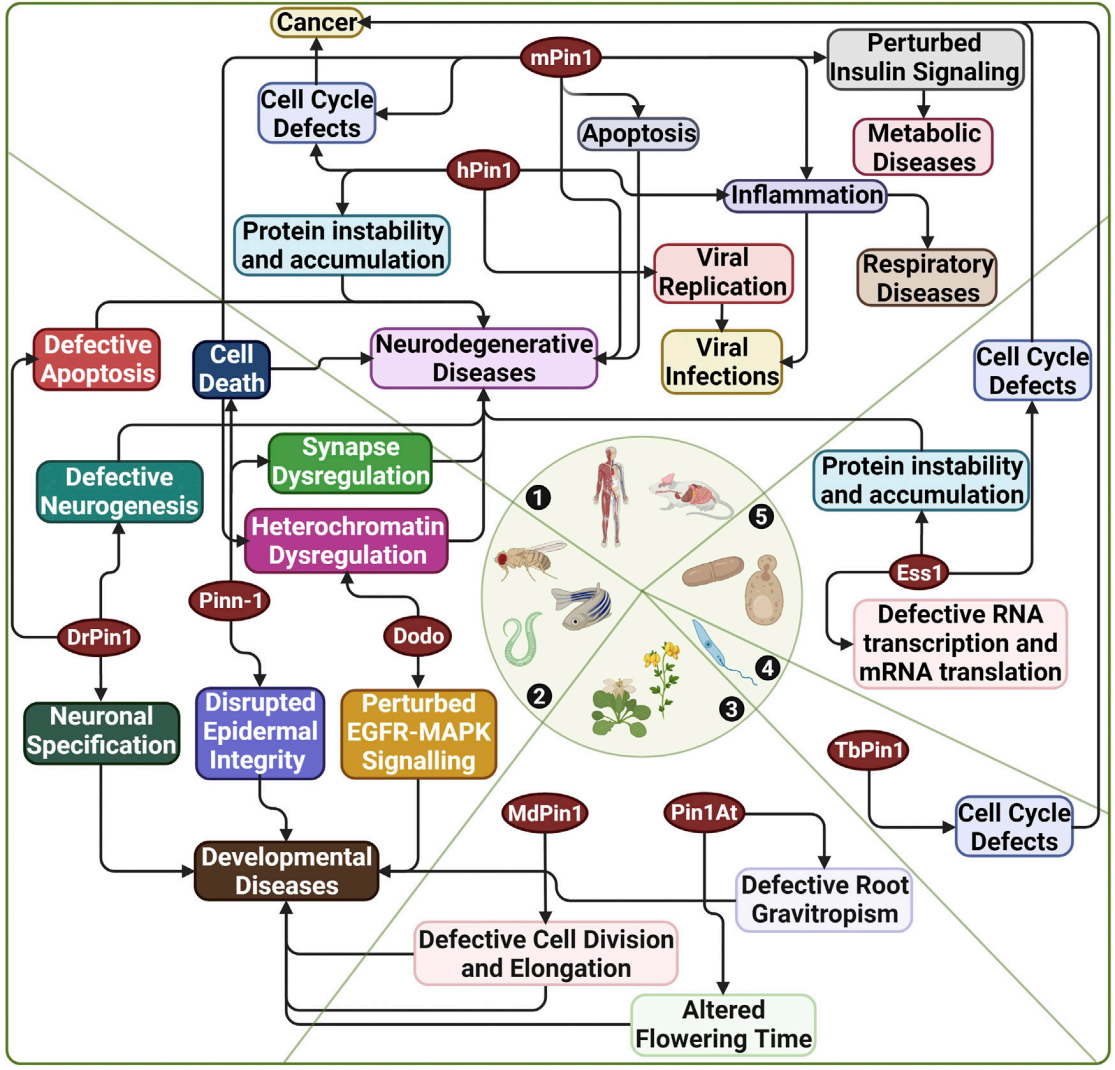
Diseases associated with Pin1 across various species. 1) Human and mouse; 2) *C. elegans*, *D. melanogaster,* and *D. rerio*; 3) A. *thaliana* and *M. domestica*; 4) *T. brucei*; 5) *S. cerevisiae*. hPin1: Human Pin1; mPin1: Mouse Pin1; DrPin1: *D. rerio* Pin1; Pinn-1: *C. elegans* Pin1; Dodo: *D. melanogaster* Pin1; Ess1: *S. cerevisiae* Pin1; MdPin1: Malus domestica Pin1; Pin1At: *A. thaliana* Pin1; TbPin1: *T. brucei* Pin1. Created with BioRender.com.

Furthermore, Pin1’s heavy involvement in diseases has made it a target for drug development. Notably, Pin1 research utilises model organisms for elucidating various functions of Pin1, modelling diseases and clinical applications in drug discovery and design. This review section will highlight Pin1’s functions and involvement in diseases and how various model organisms have implicated this research. Figure 4 highlights the extensive and interconnected role of Pin1 in disease manifestation across multiple species of which Pin1 orthologue has been identified.

Perhaps the most prominent role of Pin1 is in cancer, whereby Pin1 plays a role in many cancer pathways, which resulted in Pin1 being a drug candidate for cancer therapy. Hanes et al. (1989) first identified the function of Pin1 in cell cycle progression using yeast, as conditional mutants of Ess1 in *S. cerevisiae* displayed growth arrests and defects in the late stage of the cell cycle under non-permissive temperatures. Indeed, Pin1 regulates all stages of the cell cycle through its function in regulating phosphorylation, consistent with the fact that cell cycle regulation operates through the phosphorylation of cell cycle proteins (Lin et al., 2015; Cheng and Tse, 2018). Namely, Pin1 targets proteins such as Cdc25 (cell division cycle 25), cyclin D, and Cdk (cyclin-dependent kinase) proteins (Cheng and Tse, 2018). Additionally, Pin1 plays a role in the regulation of p53, whereby Pin1 controls the stability of p53 through direct interaction *via* the pS/T-P motifs of p53. Pin1 also leads to increased p21 expression *via* activation of p53. Since p53 and p21 function in DNA damage response and apoptosis, Pin1’s regulation of these two proteins implicates Pin1 in the pathogenesis and onset of cancer (Wulf et al., 2002; Zacchi et al., 2002). On top of regulating the wild-type Pin1, Girardini et al. (2011) demonstrated that overexpression of Pin1 regulates mutant p53 and increases cancer’s aggressiveness. Chen et al. (2018) have described how Pin1 promotes cancer by upregulating proliferative signalling, enabling cell death resistance, and enhancing metastasis and angiogenesis. Unsurprisingly, Pin1 is overexpressed in various cancers with poor prognoses (Yu et al., 2020). Mice models have been used to confirm this, wherein Pin1 transgenic mice showed tumour formation, and Pin1 knockout mice showed reduced cell proliferation (Yu et al., 2020).

Interestingly, inhibition of Pin1 increases sensitivity to chemotherapy in mice models (Zhang et al., 2020). This observation is substantiated in a study by Rustighi et al. (2014) that demonstrated the increased Pin1 expression can drive cancer stem cells (CSCs) phenotype and drug resistance in breast cancer, suggesting that the suppression of Pin1 can exhaust CSCs population and increase drug sensitivity. It is thus understandable that, over the years, researchers have attempted to target Pin1 as a treatment for cancer. Subsequently, the research community identified small molecule inhibitors against Pin1: the FDA-approved all-trans retinoic acid (ATRA) for treatment of acute promyelocytic leukaemia (Campaner et al., 2017); KPT-6566, which is selective for Pin1 and effectively targets it for degradation (Pinch et al., 2020); and sorafenib, which downregulates Pin1 expression (Zheng et al., 2017a). All in all, it is safe to say that Pin1 plays a prominent role in the formation and progress of cancer, and there have been some positive signs showing that Pin1 is a feasible target for drug therapy, as long as the drug is selective enough for Pin1.

Besides cancer, various studies also highlighted Pin1’s contribution to neurodegenerative diseases, such as Alzheimer’s disease (AD) and Parkinson’s disease (PD). In the context of AD, Lu et al. (1999a) found reduced expression of Pin1 in the brains of AD patients compared to normal brains, and the presence of Pin1 prevents and rescues phospho-tau-induced microtubule disassembly. Further research in Pin1 knockout mice revealed hallmarks of age-associated neuropathy, such as the formation of neurofibrillary tangles, loss of neurons, and deterioration of motor function, as highlighted previously. Furthermore, the study of the tau protein in these Pin1 knockout mice showed higher levels of tau phosphorylation *via* reduced activity of phosphatases that act on phospho-tau (Liou et al., 2003). Tau dephosphorylation is conformation-specific in that the PP2A phosphatase binds to the trans-pS/T-P motif of phospho-tau in a conformation-specific manner (Zhou et al., 2000). With the knowledge that PP2A is a substrate of Pin1, researchers suggest that Pin1 plays a role in regulating tau phosphorylation and, consequently, the formation of neurofibrillary tangles (Lu et al., 1999a). Biochemical and behavioural assays have further highlighted the role of Pin1 in Tau phosphorylation and neurodegeneration; hence, Pin1 knockout mice have become models to study AD (Kondo et al., 2017).

Apart from AD, Pin1 plays a role in the pathology of PD. The hallmarks of PD centre on the formation of intracellular inclusions known as Lewy bodies and the death of dopaminergic neurons in the substantia nigra of the midbrain region (Ryo et al., 2006; Ghosh et al., 2013). Expression studies of Pin1 in PD showed that Pin1 co-localises to the Lewy bodies and with Pin1 enriched in the substantia nigra of PD patient brains compared to normal healthy brains (Ryo et al., 2006; Ghosh et al., 2013). Furthermore, Pin1 overexpression led to increased formation of alpha-synuclein inclusions, while Pin1 negative mutants suppressed such inclusions. Pin1 does not facilitate the formation of Lewy bodies through direct binding with alpha-synuclein; instead, Pin1 binds *via* pS/T-P motifs to synphilin-1, and this binding promotes interaction between synphilin-1 and alpha-synuclein, which resultantly facilitates Lewy body formation (Ryo et al., 2006). Subsequent studies using mice models also demonstrated that Pin1 plays a role in the apoptotic pathway, as Pin1 knockdown cells treated with the Parkinsonism- and apoptosis-inducing drug 1-Methyl-4-phenylpyridinium (MPP^+^) showed reduced caspase activation (Ghosh et al., 2013). Besides AD and PD, Pin1 has also been implicated in other neurodegenerative diseases like Huntington’s disease (Grison et al., 2011; Agostoni et al., 2016). Taken together, studies about Pin1, particularly those done in mice models, show that Pin1’s role in the pathology of neurodegenerative diseases makes Pin1 an attractive drug target in alleviating such disorders.

In addition to cancer and neurodegenerative diseases, Pin1 is associated with viral infections and metabolic disease (Lee et al., 2009; Nakatsu et al., 2011; Narita et al., 2013; Nakatsu et al., 2015; Nakatsu et al., 2017; Nishi et al., 2020; Li et al., 2021); however, for brevity, this review will summarily highlight the role of Pin1 in these diseases. In the case of viral infections, Pin1 could positively regulate viral replication and propagation in Epstein-Barr virus (EBV), Hepatitis B virus (HBV), and Hepatitis C virus (HCV) (Lim et al., 2011; Narita et al., 2013; Nishi et al., 2020). In EBV, Pin1 binds the viral DNA polymerase catalytic subunit in a phosphorylation-dependent manner, and inhibition of Pin1 activity suppresses viral DNA replication (Narita et al., 2013). In HBV, published by Ryo’s group, Pin1 binds in a phosphorylation-dependent manner to the core protein of HBV, a similar binding pattern to EBV, stabilising the protein and preventing its lysosome-mediated degradation. When inhibiting Pin1 or blocking the phosphorylation of the core protein, the Pin1-mediated prevention of degradation was not observed (Nishi et al., 2020). Finally, in the case of HCV, Pin1 interacts in a phosphorylation-dependent manner with HC5A and HC5B, which are viral proteins that play a role in viral replication; overexpression of Pin1 leads to increased viral replication, and conversely, downregulation or inhibition of Pin1 leads to decreased viral replication (Lim et al., 2011). Besides EBV, HBV, and HCV, Pin1 is involved in other viruses such as human immunodeficiency virus (HIV) and the more recent severe acute respiratory syndrome coronavirus 2 (SARS-CoV-2) (Manganaro et al., 2010; Yamamotoya et al., 2021; Ino et al., 2022).

In the case of metabolic disorders, Pin1 regulates insulin secretion *via* regulating pancreatic beta-cell proliferation. Specifically, Pin1 interacts with SIK2 protein, which regulates Ca^2+^ levels in the pancreatic beta-cells. Knockout of Pin1 showed abnormal Ca^2+^ intracellular concentrations, impaired insulin secretion, and reduced beta-cell mass (Nakatsu et al., 2017). Furthermore, Pin1 plays a role in insulin signalling pathways, such as promoting insulin-mediated hepatocarcinoma, inducing insulin-mediated adipogenesis, and inhibiting AMPK phosphorylation and activity (Lee et al., 2009; Nakatsu et al., 2011; Nakatsu et al., 2015). Indeed, with its role in mediating insulin activities and its secretion, it is not unexpected that Pin1 plays a role in obesity and diabetes, as mentioned by Nakatsu et al. (2017). In addition, a study by Paneni et al. (2015) using diabetic mice, they demonstrated that mPin1 is upregulated by hyperglycaemia. The increased level of mPin1 is involved in molecular events that trigger the diabetic vascular disease. The knockout of mPin1 in the diabetic mice prevented vascular dysfunction. The authors went on to demonstrate that diabetic human patients have higher Pin1 expression, which correlated with the deleterious vascular phenotype and targeting Pin1 could improve vascular health. This study further highlights the role of Pin1 in metabolic diseases.

A large bulk of research on the association of Pin1 with various diseases is done on human and mouse models. However, Pin1 orthologs from other species have demonstrated their links to diseases shown in human and mice models (Figure 4). For example, Pin1 orthologs from *D. rerio* (DrPin1), *C. elegans* (Pinn-1) and *D. melanogasta* (Dodo) have been linked to neurodegenerative diseases (Del Rosario et al., 2015; Ibarra et al., 2017; Salzberg et al., 2020; Napoletano et al., 2021). Moreover, DrPin1, Pinn-1, and Dodo perturbation could also affect the development processes of their respective species (Hsu et al., 2001; Liou et al., 2002; Balastik et al., 2015). A recent excellent work published by Del Sal’s group demonstrated that Dodo preserved tissue homeostasis in *Drosophila*. Dodo/Pin1 may play a critical role in maintaining the nuclear Lamin-B structure and function and further protects heterochromatin during ageing or undergoing mechanical stress (Napoletano et al., 2021). The authors also identified such a phenomenon in mice, further highlighting the conservation of Pin1 function in heterochromatin dysregulation across species. We have also previously shown that DrPin1 knockdown led to developmental delay in the *D. rerio* embryos (unpublished). The loss of DrPin1 led to the loss of neuromast hair cells. We then identified that DrPin1 could interact with neuroD (Nrd) at all its pS/T-P motifs, with the loss of Pin1 binding to Nrd leading to Nrd degradation. In addition, Pin1 orthologs in *M. domestica* (MdPin1) and *A. thaliana* (Pin1At) could also affect plant developments such as fruit development, root growth and flowering (Yao et al., 2001; Wang et al., 2010; Xi et al., 2016). A study in 2017 by Zheng et al. (2017b) also identified a Pin1 orthologue (PvPin1) in the bamboo *Phyllostachys violascens*. The authors showed that PvPin1 overexpression causes the delay of flowering time in *A. thaliana* and the rice *Oryza sativa*. While in *T. brucei*, TbPin1 regulates cell growth, as demonstrated previously by our laboratory (Goh et al., 2010).

It is evident that Pin1, with its vast array of molecular targets, is involved in many diseases and seems to be interconnected among various species. Indeed, it is highly worthwhile to study Pin1’s role in diseases as it may prove to be an effective drug target, as shown in the example of cancer (Campaner et al., 2017; Pinch et al., 2020). It is important to note that Pin1’s role in diseases can be studied through *in vitro* assays or diseased animal models. While *in vitro* studies have many advantages, they cannot observe the organism-level complexity of disease progression and drug design. There are vital aspects of research that must be done using animal models. It is thus essential to note the critical variations between hPin1 protein and its orthologs in other organisms. However, an important thing to note is that perturbation to Pin1 orthologs leading to various disease manifestations might be species-specific due to the differences in signalling pathways and biological processes, such as between plants and humans. Therefore, despite highlighting the potential similarities of Pin1 orthologs in disease manifestation across species, care has to be taken not to over-extrapolate the findings. Nonetheless, such information would be helpful in our quest to identify better ways to target Pin1 in various diseases.

## Future perspectives

This review has highlighted that Pin1 protein is a highly conserved protein, with hundreds of orthologous Pin1 species (National Library of Medicine (US), 2004). Indeed, Pin1 orthologs in model organisms have given insights into specific mechanisms and disease phenotypes that are impossible to study in human experiments. However, it is essential to be mindful of the structural and functional variations between hPin1 and its orthologs in other species; thus, extensive characterisation of such cross-species variations helps generate better models for human Pin1. As mentioned in an earlier section of this review, further research on the structure of Pin1 orthologs upon binding to the substrates may give a greater insight into binding mechanisms and aid drug discovery. In addition, the differences in sequence and structure of Pin1 orthologs could allow them to bind and interact with an increased variety and repertoire of Pin1 substrates/interactors. This increased number of Pin1 substrates/interactors could suggest a higher level of sophistication in Pin1 regulation of their phosphorylated Pin1-binding sites, thus explaining their differences in Pin1 function across species. Therefore, understanding these differences in Pin1 orthologs would further our understanding of the variation of Pin1 mechanisms, which could prove helpful in generating a new generation of Pin1 inhibitors.

Notably, much progress has been made in Pin1 disease research and therapy, as seen in the case of the FDA-approved anti-cancer drugs, ATRA, arsenic trioxide, and sorafenib (Zheng et al., 2017a; Campaner et al., 2017; Kozono et al., 2018). However, the successes of these drugs are limited (Coombs et al., 2015; Cheng and Tse, 2019; Chuang et al., 2021). Indeed, Pin1 has been a difficult drug target because its active site is shallow and highly conserved. Although highly electronegative compounds could bind to the active site of Pin1, the increased negative charge could reduce the cellular membrane permeability of the Pin1 inhibitor (Dunyak and Gestwicki, 2016). Nonetheless, successes in using Pin1 inhibitors in combination with other therapies show much hope in overcoming the limitations of single-agent Pin1-inhibitor drugs (Zheng et al., 2017a; Koikawa et al., 2021). Recently, a review by Chuang et al. (2021) described the current scope of Pin1 targeting comprehensively in cancer and highlighted certain aspects to improve efficacy and delivery, including increasing the bioavailability of Pin1 inhibitors and improving the water solubility of Pin1 inhibitor drugs. Additionally, this review suggests that more research can be done to incorporate model organisms in drug discovery. The high conservation of Pin1 could be used as an advantage in drug discovery and development, for example, in developing better delivery systems and validating Pin1 as a drug target.

Besides the issue with cell permeability, the current selection of Pin1 inhibitors is non-selective. For example, the Pin1 inhibitor, juglone, is known to have off-targets such as RNA polymerase II (Chao et al., 2001). ATRA, another known inhibitor of Pin1, interacts with retinoic acid receptors besides being able to degrade Pin1 (Wei et al., 2015). The more recently identified Pin1 inhibitor, KPT-6566, releases a quinone-mimetic substructure upon binding with Pin1 (Campaner et al., 2017). This quinone-mimetic substructure can interact with many other proteins to induce an oxidative stress response. In 2020, Pinch et al. (2020) identified a new potent and selective Pin1 inhibitor, BJP-06-005-3. BJP-06-005-3 covalently binds to Cys113 of Pin1, leading to Pin1 degradation. Despite the promising potency and selectivity of BJP-06-005-3, further research has to be done to understand the efficacy of BJP-06-005-3 better. Furthermore, BJP-06-005-3 can be used as a tool to better understand Pin1 orthologs biology in diseases across various species.

On top of finding covalent inhibitors of Pin1, the uniqueness of the *cis/trans* isomerisation of protein by Pin1 has been explored previously to generate conformation-specific antibodies by Nakamura et al. (2012). The authors also found that *cis* pT231-tau is more pathogenic in AD, with 90% of regular synthetic pT231-tau peptides existing in *trans* configuration. They suggest that developing conformation-specific vaccines could be a more effective way to target Pin1-induced diseases, along with therapeutic conformation-specific antibodies against Pin1 targets. Indeed, as we investigate the different Pin1 orthologs across various species and diseases, using such conformation-specific antibodies could be a new area to explore in targeting Pin1-associated diseases.

In conclusion, this review proposes two main areas of further Pin1 research: firstly, improving the structural and functional understanding of Pin1 orthologs, and secondly, utilising model organisms more extensively and effectively in the drug development process. Indeed, these two points are highly linked because as the understanding of Pin1 orthologs in model organisms grows, such research can be used more effectively in drug development. Conversely, as model organisms are utilised in Pin1-targeting drug development, the gaps in the current literature on Pin1 orthologs are exposed, and research can be done to fill them. In addition, an area of interest lies in the structural difference between single domain Pin1 found prominently in Pin1 orthologs from plants and the double domain Pin1. As highlighted in our previous review on the potential significance of interdomain interaction in Pin1 function, it is noteworthy whether the absence of the interdomain interaction in single domain Pin1 orthologue would have implications for their role in diseases (Lee and Liou, 2018).
